# Lattice Genome Framework for Regionally Tailored Component‐Level Multi‐Objective Design in Additive Manufacturing

**DOI:** 10.1002/advs.202522126

**Published:** 2026-02-15

**Authors:** Haoyuan Deng, Yufan Zhao, Mingyang Cao, Haiou Yang, Pan Wang, Hongze Wang, Hao Wang, Akihiko Chiba, Xin Lin

**Affiliations:** ^1^ State Key Laboratory of Solidification Processing Northwestern Polytechnical University Xi'an P. R. China; ^2^ Key Laboratory of Metal High Performance Additive Manufacturing and Innovative Design MIIT China Northwestern Polytechnical University Xi'an P. R. China; ^3^ Singapore Institute of Manufacturing Technology (SIMTech) Agency for Science Technology and Research (A*STAR) Cleantech Loop Republic of Singapore; ^4^ School of Materials Science & Engineering Shanghai Jiao Tong University Shanghai P. R. China; ^5^ Co‐Creation Institute for Advanced Materials Shimane University Matsue Shimane Japan; ^6^ New Industry Creation Hatchery Center (NICHe) Tohoku University Sendai Miyagi Japan

**Keywords:** additive manufacturing, lattice structures, materials genome initiative, mechanical properties

## Abstract

Lattice structures fabricated by additive manufacturing provide unprecedented opportunities for lightweight design, mechanical regulation, and functional integration. However, their efficient development remains constrained by vast design spaces and complex structure–property relationships. Inspired by the Materials Genome Initiative, we propose a Lattice Genome framework, a data‐driven approach that integrates high‐throughput simulations and performance databases to systematically map lattice properties. Specifically, we develop a component‐level, regionally programmable, multi‐objective design strategy for coordinated structure–property regulation. The efficacy of the proposed framework is demonstrated through two representative cases: (1) tailoring internal stress distributions to match preset targets under constant relative density, and (2) enhancing load capacity (62% higher than the conventional design) while redirecting failure away from critical zones in cavity‐containing components. Overall, this work establishes a generalizable and scalable paradigm for intelligent lattice design and provides a transferable, data‐centric platform that lays the groundwork for efficient, multifunctional applications in additive manufacturing.

## Graphical abstract

A Lattice Genome framework links geometric and process “genes” to lattice “phenotypes” via correction‐calibrated high‐throughput simulations and a growing performance database. Genome‐driven retrieval and recombination of unit cells enables component‐level, regionally tailored multi‐objective design: stress fields are programmed under constant relative density, and failure is steered away from protected cavities while boosting load capacity.

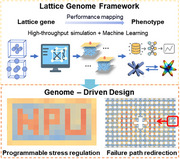

## 1 Introduction

Lightweight design has long been a main topic in aerospace engineering, driving the exploration of novel materials and advanced structural concepts. Although many lightweight materials have been proposed, their performance is still limited by their own material properties [1]. In recent years, architected materials, particularly porous lattices, have gained increasing attention because they have outstanding mechanical properties, low weight, and high design flexibility [2, 3]. Lattice structures are a typical type of architected material. They combine stiffness, strength, and energy absorption, rendering them valuable for aerospace, biomedical, and mechanical engineering [4, 5, 6, 7]. With the advent of advanced additive manufacturing technologies, the integration of complex lattice geometries into functional components has been accelerated [8]. Among these methods, the powder bed fusion with laser (PBF‐L) is regarded as the most suitable for fabricating metallic lattices [9, 10, 11].

Research has shown that the lattice topology, unit cell geometry, and combination of multiple lattices within a single component significantly influence the mechanical, thermal, and other integrated properties of additively manufactured parts [12, 13, 14]. However, this high level of design freedom also makes the structure–property relationship very complex [15], rendering traditional design methods based on experience no longer enough. Among representative studies, Guo et al. enhanced the stress uniformity and plastic strain capacity of components by embedding reinforcing topologies to form dual‐topology unit cells [16]. Jadhav et al. applied a conditional denoising diffusion probabilistic model to inverse‐design triply periodic minimal surface structures, generating unit cells with high energy absorption and compressive load capacity [17]. Jin et al. proposed a mapping‐based self‐generating multiscale modeling method that improved modeling efficiency and accelerated mechanical evaluation [18]. Notably, these approaches focus on small‐scale lattice structures and can facilitate only local optimization. By establishing systematic datasets, these approaches could be extended to support large‐scale lattice and component‐level design.

Lattice components in practical engineering applications are often subjected to complex service conditions and demand a high degree of integration. Consequently, multi‐objective co‐optimization has emerged as an important direction in component design [19, 20], drawing increasing attention to regional performance regulation. Multi‐objective regional performance regulation involves the spatially differentiated configuration of lattice unit cells according to the local loading state or functional requirements of different regions within a component, thereby achieving coordinated optimization of the overall performance and local mechanical response. For metallic lattices, researchers have attempted to regulate regional performance by controlling PBF‐L laser power and scanning speed [21, 22], constructing graded relative densities [23, 24], or applying machine‐learning approaches [25, 26]. However, the range of achievable performance regulation remains limited by the available lattice types with known properties and their manufacturability.

Many existing lattice design workflows have achieved substantial improvements in component‐level performance, and related studies can be broadly categorized into three representative directions. First, machine‐learning‐based lattice design approaches have primarily focused on unit‐cell performance prediction, inverse design, and indirectly enhancing component performance through optimized unit‐cell design [27, 28]. These methods offer clear advantages in accelerating design‐space exploration. However, their training data are typically derived from idealized geometries, and a complete mapping from design parameters to manufacturing‐induced deviations and ultimately to experimental performance has yet to be established. In other words, they lack a unified linkage spanning geometric “genotypes” and structural “phenotypes,” which makes it difficult to impose physical constraints and hinders their direct extension to component‐scale regional performance regulation.

Second, approaches that combine topology optimization with lattice structures generally employ global objective functions as the primary driving mechanism. Their main advantage is that they combine the field‐based idea of continuum topology optimization with lattice infilling. This makes it possible to greatly reduce weight and still keep good mechanical performance. It also provides a repeatable optimization method for components with complex boundary conditions. Nevertheless, these methods often focus on only a few design objectives [29], and they usually aim at a specific performance measure, such as stiffness [30, 31], strength [32], or energy absorption [33]. As a result, the optimized outcomes are highly sensitive to boundary conditions and loading cases, and it is difficult to achieve independent control in different functional regions. Moreover, topology‐optimized results usually require extensive post‐processing to be converted into manufacturable lattice structures, and their manufacturability and component‐scale closed‐loop experimental validation remain challenging, which limits their direct applicability in engineering practice.

Third, multi‐objective functionally graded lattice design methods achieve spatially varying performance by continuously adjusting relative density or unit‐cell geometric parameters. Their strength lies in constructing smooth performance fields with relatively low design complexity and in partially accommodating multiple objectives, such as balancing stiffness, strength, and energy absorption. However, because these methods fundamentally rely on continuous gradient assumptions and a small number of scalar design fields, they struggle to provide precise and independently controllable regulation for regions with clearly defined functional roles (e.g., load‐bearing, buffering, or protection zones). Furthermore, when components face complex multi‐objective fields or need many functions, designers often have to balance competing objectives, making it difficult to rapidly find effective regionally tailored lattice‐filling schemes for different regions without greatly increasing the total design cost [14, 18].

These three types of lattice component design and optimization methods each exhibit their own strength. But they also have challenges such as high computational cost, limited application adaptability, and insufficient global optimization capability [34]. Consequently, systematic design approaches that allow fine control, easy scaling, and reuse across many regions at the component level are still rare. Therefore, a new design method is needed to change separate local optimizations into a data‐driven, global, and programmable framework [19, 34].

To solve these challenges, this study introduces a Lattice Genome framework for additive manufacturing. It establishes a complete mapping from geometric “genotypes” to structural “phenotypes.” This helps researchers study the broad lattice design space in a systematic way and develop a component‐based, multi‐objective design method based on the lattice genome. This idea is directly inspired by the Materials Genome Initiative proposed in 2011, whose core principle is to accelerate paradigm shifts in design through data and high‐throughput computation [35]. The Lattice Genome framework consists of two main parts: high‐throughput simulation and lattice‐performance database construction. Two representative case studies show how well this framework works: (1) Under the same relative density constraint, the stress distribution within a loaded component can be adjusted to match preset targets; and (2) under the same relative density constraint, for a component with a reserved cavity for sensitive devices, the stress during loading becomes more uniform, and the overall load capacity improves; moreover, the failure initiation site moves away from the critical region.

Overall, this study not only proposes a novel research framework for lattice structures but also shows its potential for multi‐objective design, including the joint control of relative density, stress distribution, and controllable failure location. It provides a general paradigm for discovering intelligent metamaterials in different material systems and for engineeringfunctional structures under diverse service conditions.

This paper first presents the workflow of the Lattice Genome framework. It then shows typical lattice data obtained within this framework and explains the workflow components related to simulation accuracy correction and machine learning prediction. It then demonstrates two application cases built upon this framework. Subsequently, the advantages and limitations of the work are discussed, and directions for future research are outlined. Finally, the paper provides the specific methods for implementing lattice genome exploration and programmable design of engineering components.

## 2 Results

**FIGURE 1:**
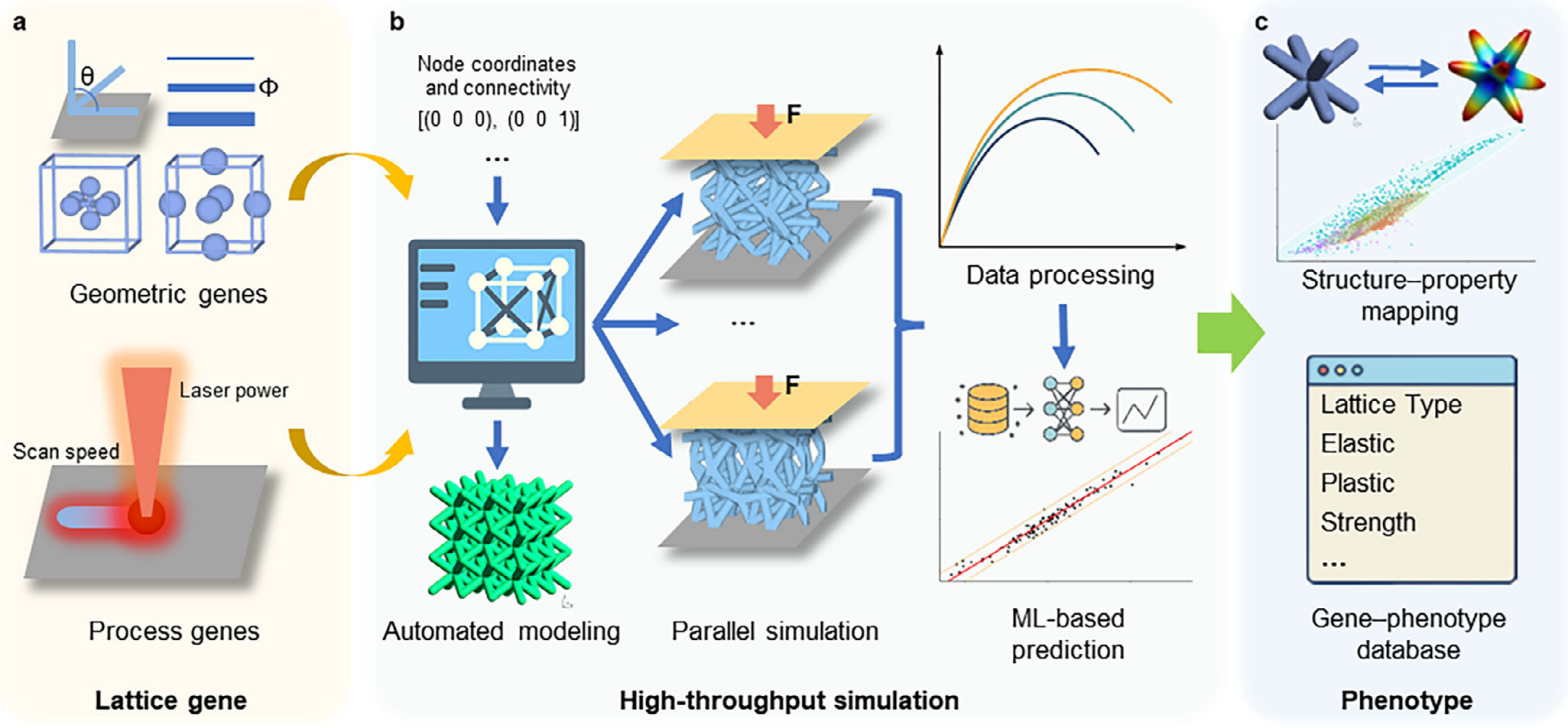
Workflow of the Lattice Genome framework for additive manufacturing. (a) Lattice genotypes consisting of geometric and process genes. (b) High‐throughput simulations including automated modeling based on node coordinates and connectivity, parallel finite‐element analysis, automated data post‐processing, and training of machine‐learning surrogate models for performance prediction. (c) Structure–property mappings established from high‐throughput simulation data to construct a genotype–phenotype database.

The proposed framework is established drawing inspiration from the Materials Genome Initiative. Figure 1 illustrates the basic workflow of the Lattice Genome framework for additive manufacturing. Within this framework, a “gene” is defined as the minimal unit of lattice design that can be encoded, reused, and spatially recombined. Lattice genes consist of two core entities (Figure 1a): geometric genes (unit size, unit topology, relative density, strut morphology correction, etc.) and process genes (material constitutive parameters, process parameters, and equivalent properties calibrated under manufacturing conditions). The former determines geometry and load paths, whereas the latter captures the influence of additive manufacturing on mechanical responses. The proposed Lattice Genome framework is built on the central concept of genotype–phenotype mapping, which can be decomposed into four complementary stages: random generation of multiple lattice types under geometric constraints; high‐throughput finite‐element simulations calibrated by geometric and process parameters to obtain phenotypic properties in batches, followed by machine learning to construct predictive models and train rapid genotype‐to‐phenotype predictors (Figure 1b); establishment of structure–property gene maps and continuous expansion of a lattice gene database (Figure 1c); and, finally, use of the database and predictors to recombine genes and regulate performance according to preset target fields, enabling genome‐driven component‐level design for specific applications.

While researchers have identified many high‐performance lattices under various loading conditions [36, 37, 38, 39] and controlled stress distribution by adjusting relative density or cell type, reports of multi‐objective joint regulation remain scarce. Notably, in real engineering applications, components are often subjected to coupled loads and multiple constraints, rendering single‐objective optimization insufficient. Thus, multi‐objective joint regulation is crucial for lattice design under complex service conditions.

### 2.1 High‐Throughput Simulation: Core Data Engine of the Lattice Genome Framework

Unlike traditional lattice design approaches based on theoretical derivations [40], trial‐and‐error, topology optimization, [41], or multistructure nesting [42], this study employs fully automated high‐throughput simulations to explore a large‐scale design space. With only a few geometric constraints specified, the program can rapidly generate tens of thousands of configurations and evaluate their mechanical properties in parallel. Rather than identifying so‐called “optimal structures” sequentially, the proposed framework emphasizes the construction of a comprehensive dataset in which each topology becomes a data point within the structure–property mapping. This strategy removes human bias and ensures the design space is both complete and diverse.

**FIGURE 2:**
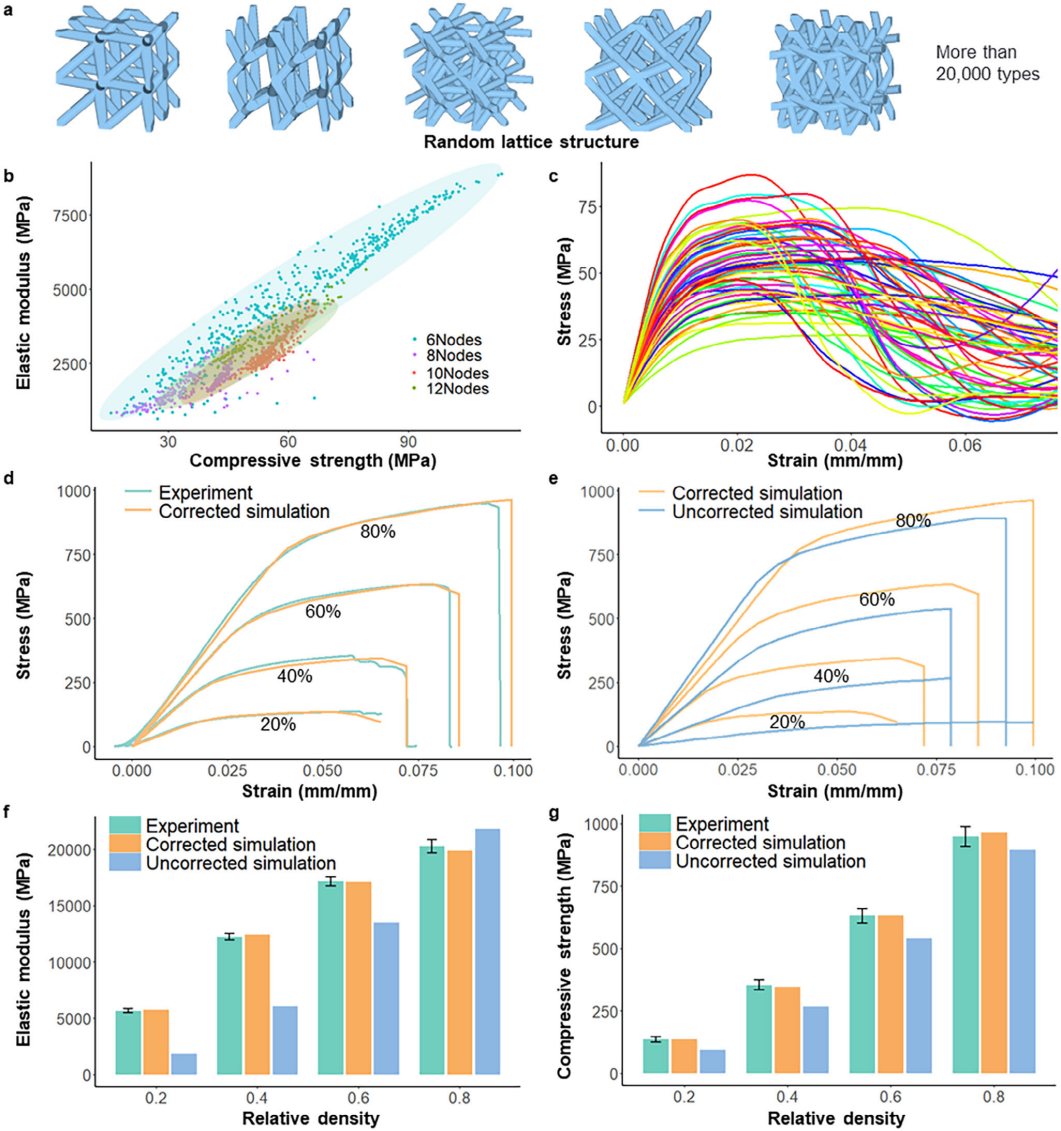
Compressive mechanical properties of random lattice structures and comparison between experiments and simulations at multiple relative densities. (a) Randomly generated lattice structures. (b) Elastic modulus and compressive strength of lattices categorized by the number of common nodes. (c) Stress–strain curves of representative lattices. (d) Comparison between experiments and corrected simulations. (e) Comparison between corrected and uncorrected simulations. (f) Elastic modulus comparison. The error of uncorrected simulations is particularly pronounced at low relative densities, exceeding 50%. (g) Compressive strength comparison.

During random generation, only basic geometric constraints (e.g., PBF‐L manufacturability) are set. Other geometric attributes are randomly combined by the program. This prevents subjective performance preferences and lets the database include many different lattice types. All generated structures are added to the structure–property mapping without “good” or “bad” labels, so data integrity and general use are maintained. The number and locations of common nodes directly decide whether different lattices can be assembled together. This ensures geometric continuity and improves the feasibility of large‐scale component design and automated filling. When the results are grouped by the number of common nodes, the mechanical properties exhibit clear distribution patterns (Figure 2b). This reveals implicit statistical correlations between geometric features and overall performance. Under a fixed relative density, the total material volume in each unit cell remains constant. Increasing the number of common nodes enhances structural connectivity, which stabilizes load transfer and reduces response dispersion. However, higher connectivity is typically accompanied by an increase in the number of struts, causing the limited material volume to be distributed among more load‐bearing members and thereby reducing the effective load‐carrying cross‐sectional area of individual struts. In addition, additive manufacturing is subject to a minimum manufacturable feature‐size constraint; as the strut diameter decreases and approaches this process limit, the range of admissible strut diameters after manufacturability screening is effectively compressed.

Under the Z‐axis compression conditions considered in this study, the peak load is generally governed by axially loaded critical struts, whose failure is primarily determined by their minimum effective cross‐sectional area and slenderness ratio. Consequently, an increased number of common nodes may promote earlier buckling instability, which competes with the load‐sharing benefits associated with higher connectivity. This dual effect—namely, the restriction of the admissible strut‐diameter range and the trade‐off between connectivity and strut‐level strength—collectively gives rise to the performance bands observed in the figure that are dependent on the number of common nodes. This shows the potential of the genome database in revealing structure–property relationships.

Through this workflow, more than 20 000 random lattice compression simulations were completed in 5 months on a single workstation (Intel Xeon Gold 6430). From these simulations, the elastic modulus and compressive strength were extracted (Figure 2). At a relative density of 20%, the performance range was 1000–10 000 MPa for the elastic modulus and 20–120 MPa for compressive strength. This represents a very broad design space.

This dataset constitutes the prototype of a Lattice Genome database. It provides large training samples for subsequent machine learning predictions and shows statistical relationships between structural geometric parameters (e.g., number of common nodes) and macroscopic mechanical properties. This genome‐driven approach enables systematic knowledge growth and transfer in lattice design, and provides a base for programmable, multi‐objective regulation.

### 2.2 Simulation Correction for Enhanced Accuracy

A long‐standing bottleneck in lattice design based on finite element simulations is the systematic deviation between numerical predictions and experimental observations, which primarily arises from manufacturing‐induced effects in the PBF‐L process—specifically, the staircase effect that causes inclination‐dependent variations in as‐built strut diameters, as well as the size‐dependent degradation of mechanical properties at small characteristic dimensions [43, 44, 45]. Existing correction methods attempt to reduce such errors by adjusting local stiffness, changing node diameters, or adding defect features [46, 47, 48]. However, these approaches usually focus on local compensation for a single lattice type and lack generalizability for large groups of structures.

To solve this problem, this study adopts a two‐stage simulation correction strategy (Figure S4). First, the designed strut diameter is mapped to an effective load‐bearing diameter based on the build inclination angle. This describes geometric deviations caused by manufacturing, and the lattice geometry model is updated accordingly. Second, an experiment‐based nonlinear mechanical correction approach is introduced. The effective constitutive parameters in the finite element model are adjusted to reflect the drop in mechanical performance caused by diameter changes. This correction does not imply the existence of intrinsic size‐dependent mechanical behavior of the material. It serves as an effective calibration procedure aimed at reducing the discrepancy between simulations and experiments. Unlike conventional “empirical compensation” methods, this approach can be directly embedded into the high‐throughput simulation workflow and keep stable correction accuracy for different lattice topologies and size ranges.

Figure 2d–g compares the experimental results with the uncorrected and corrected simulation predictions. After applying the proposed correction strategy, the average deviations between simulated and experimental elastic modulus and compressive strength across different relative densities are reduced to below 5%. These results demonstrate that the correction strategy enables reliable and scalable acquisition of lattice mechanical properties within a high‐throughput simulation framework.

### 2.3 Machine‐Learning Prediction of Lattice Performance

To evaluate the role of the database in performance prediction, an ensemble learning model was trained using lattice geometric parameters as inputs and elastic modulus and compressive strength as outputs. Figure S1 presents the prediction performance of the machine‐learning model, including learning curves during hyperparameter tuning (Figure S1a,b,d,e) and comparisons between predicted and true values (Figure S1c,f). The model achieved high prediction accuracy on the test set: For the compressive strength, the coefficient of determination and mean squared error were *R* ^2^ = 0.92 and MSE = 8.86 MPa^2^, respectively. The corresponding values for the elastic modulus were *R* ^2^ = 0.91 and MSE = 1.51 × 10^5^ MPa^2^. Overall, the deviation between predicted and actual values was controlled within 5%, demonstrating strong consistency between predicted and actual performance.

These results indicate that the large‐scale database constructed from high‐throughput simulations can support reliable prediction of key properties across different lattice structures using machine learning. Conventional approaches depend on empirical formulas or predictions for only one lattice type. The data‐driven method in this study can capture and generalize structure–property relationships in a systematic way and apply them to many structures. Such predictability shows that lattice performance can be programmed. It also provides a solid computational foundation for subsequent regional regulation and multi‐objective optimization.

### 2.4 Application Case I: Programmable Stress Regulation Under Constant Relative Density

Conventional stress regulation strategies typically rely on combining a limited number of unit types or constructing graded relative densities. For example, Jia et al. [49] modulated stress through different combinations of four types of basic building blocks, but the range of tunability was limited. Chen et al. [50] and Takezawa et al. [51] modulated performance by combining unit cells of different relative densities. While such strategies improve local performance, they alter the overall mass distribution, leading to shifts in the center of gravity and inertia characteristics. This constrains higher‐level optimization in large‐scale engineering applications where global modal properties or stability must also be considered.

**FIGURE 3:**
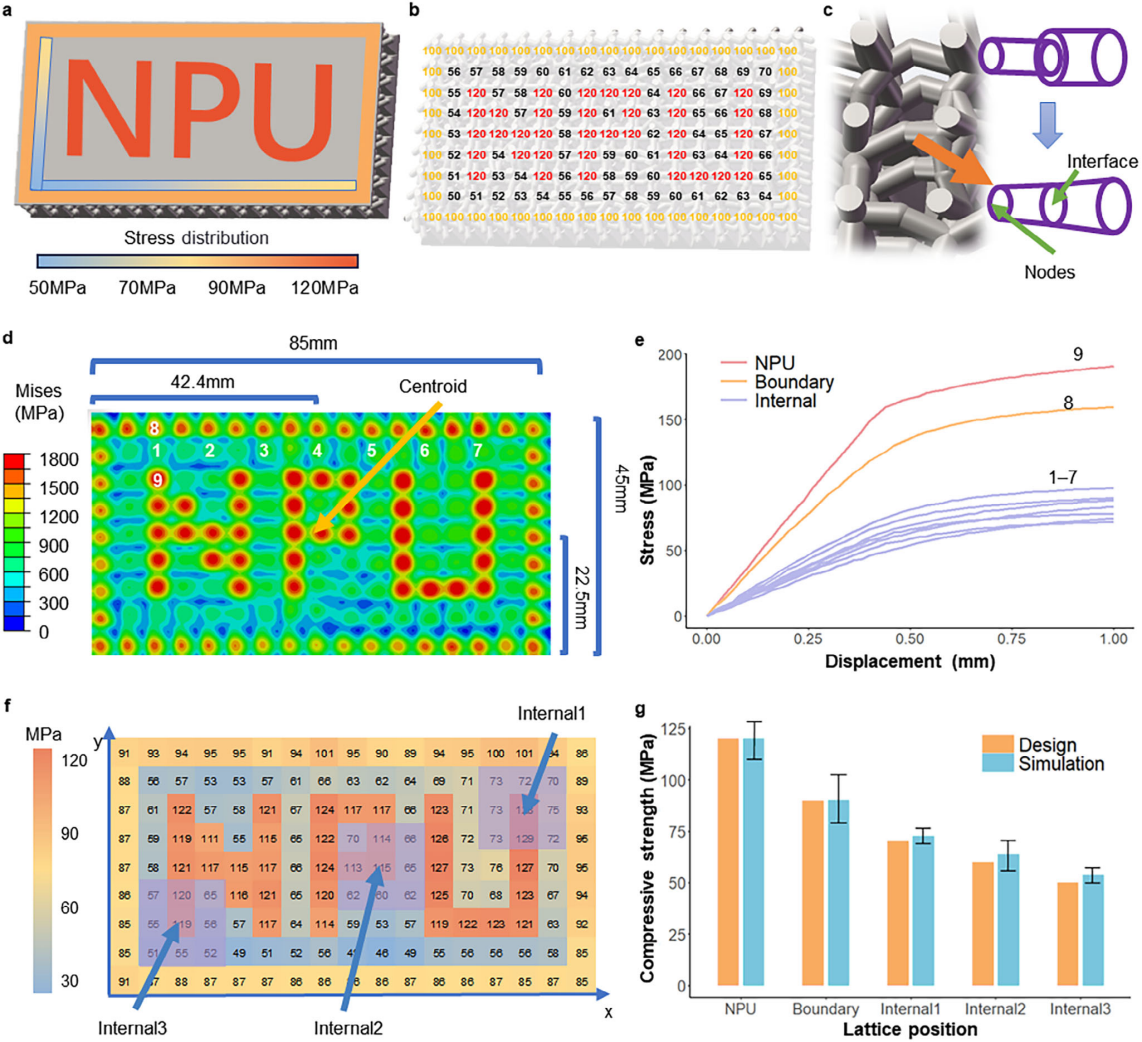
Design and performance of a component with programmable stress regulation. (a) Schematic of the component design objectives. (b) Design matrix of the component. (c) Interface design between different lattices. (d) Finite element simulation results. (e) Stress–displacement curves of nine key nodes. (f) Average stress of surface regions categorized by lattice domains. (g) Comparison between designed and simulated values at three characteristic locations.

The present study is the first to demonstrate that within the proposed Lattice Genome framework, the internal stress distribution can be programmably regulated while maintaining constant overall relative density. Specifically, a cuboidal component was constructed and divided into three functional regions (Figure 3a): The key letter region was embedded with lattices exhibiting the highest compressive strength; the border was filled with lattices of the next‐highest strength; and the interior region was populated with 69 lattices of progressively increasing strength. Some of these designs came from high‐throughput finite element simulations and partly from machine learning predictions, yielding a total of 71 distinct lattices that enabled differentiated filling (Figure 3b). To ensure seamless different lattices connected smoothly, conical transition structures were introduced at the interfaces, with diameter differences controlled within 0.1 mm (Figure 3c). This kept the overall relative density and macroscopic geometry, ensuring that the center of gravity of the component remained consistent with that of the corresponding solid. This helped prevent additional perturbations to mechanical performance. The entire process of modeling and boolean merging was fully automated. This shows the efficiency and scalability of the Lattice Genome approach.

The finite element simulation results further verified the effectiveness of this approach. Under a 1 mm compressive displacement, the internal stress distribution of the component closely matched the design targets. The letter region had the highest stress, the border came next, and the interior displayed a smooth gradient (Figure 3d). The stress–displacement curves of key nodes also revealed pronounced regional differences in load‐bearing capacity (Figure 3e). The deviations between the average stress in each functional region (defined by lattice unit domains) and the design targets were within 10% (Figure 3f,g). This also indicates that, when homogenized at the unit‐cell level, the deviation between conventional simulations and our genome‐based preset predictions is negligible, enabling the framework to effectively replace full‐scale simulations and substantially improve computational efficiency. Traditional methods based on density gradients or unit‐cell topology combinations offer only a small tuning range and lack quantitative clarification of their regulatory boundaries. This range directly determines the engineering applicability of the method. In this study, the differentiated filling strategy achieved a tunable stress range of up to 100 MPa at a relative density of 20%. The database includes many common‐node lattices with low boundary effects; this approach ensured high continuity, connectivity, and wide performance coverage.

The main contribution of this case is the creation of a programmable method for stress distribution regulation under a constant relative density. The genome‐driven differentiated filling improves the accuracy and predictability of stress regulation and shifts component design from local unit optimization to global coordinated optimization. This methodological breakthrough lays the groundwork for multi‐objective regulation and can be extended to more complex multiphysics scenarios such as impact response, energy absorption, and thermal management. It also shows strong potential for scalable component‐level additive manufacturing.

### 2.5 Application Case II: Enhanced Load‐Bearing Capacity and Failure Path Redirection

In engineering systems such as electronic compartments in aerospace components or battery modules in energy systems, functional zones often need special protection. Traditional uniform lattice‐filling schemes tend to fail first in the core region owing to structural changes and strength reduction, eventually causing damage to the embedded devices and leaving almost no buffer for mitigation. Previous research on damage‐programmable design has cleverly exploited nature‐inspired crack‐resisting mechanisms to advance the control of failure pathways and fracture resistance [52]. This study further demonstrates, within a Lattice Genome framework, the differentiated filling approach that enhances load‐bearing capacity and deliberately redirects failure sites while maintaining constant relative density and global geometry, and offers broader prospects for extension.

**FIGURE 4:**
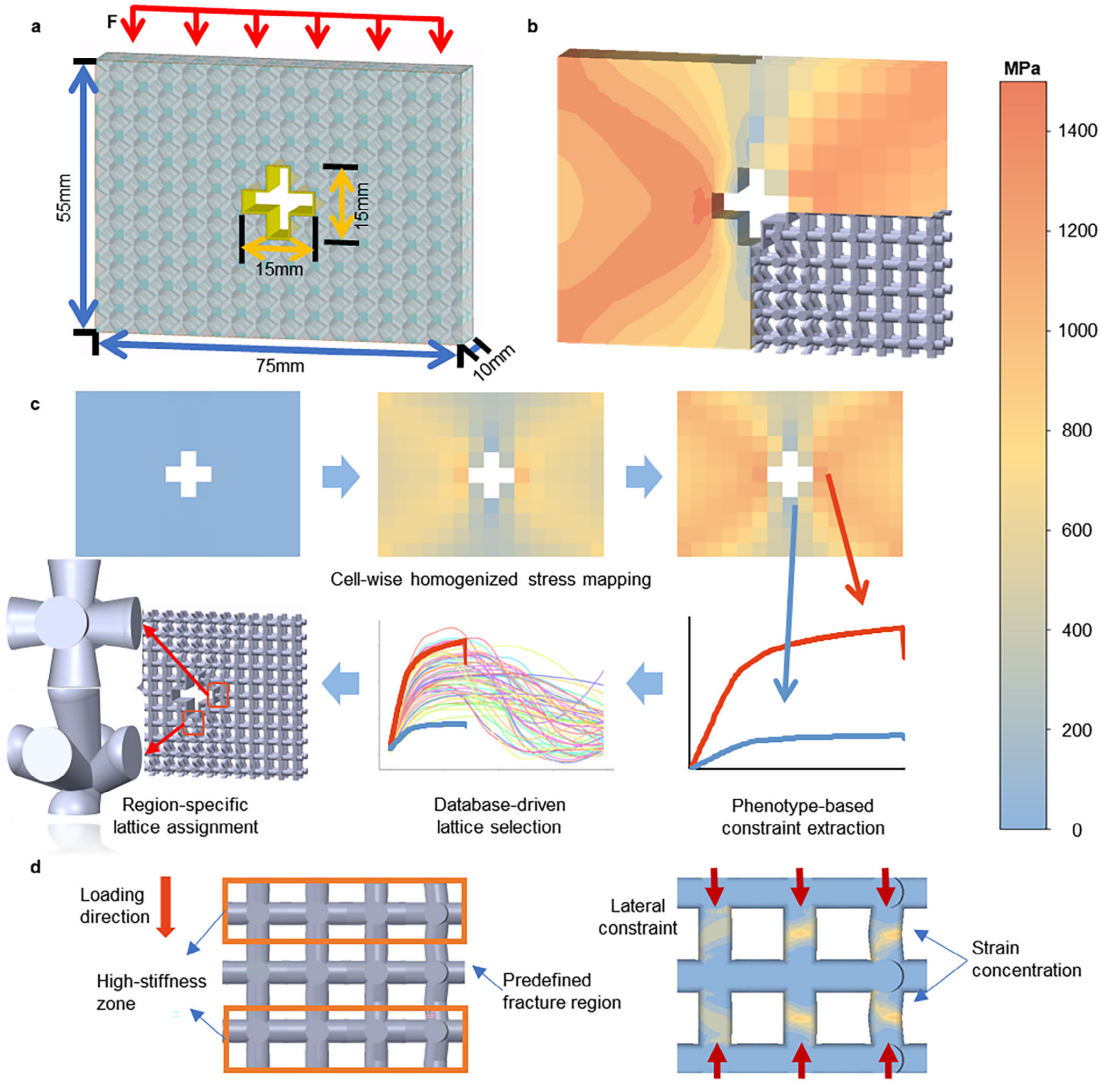
Design workflow of regional performance regulation in programmable lattice structures. (a) Geometry of the specimen and boundary conditions: A vertical compressive load is applied at the top; the interior is filled with lattices; and a central cross‐shaped cavity is preset, representing a critical region. (b) Stress distribution obtained from finite element simulations, with stresses homogenized over each lattice domain (5 mm × 5 mm × 5 mm) and used to guide lattice filling. (c) Lattice‐filling strategy, where stress–strain curves are extracted for each lattice domain, and matched lattices are retrieved from the performance database and assigned to corresponding locations. (d) High‐stiffness layers are placed on both sides of the predefined fracture region to direct strain into the middle layer and trigger earlier failure.

In this analysis, a functional component with a central cavity was used as the model system (Figure 4a), with the cavity representing a critical region requiring protection. To compare different design strategies, three schemes were constructed: (1) uniform body‐centered cubic (BCC), in which the entire component was filled with the same unit cell; (2) global strongest uniform maximum lattice (UML), in which all regions were filled with the lattice exhibiting the highest load‐bearing capacity in the regionally programmable lattice structure (RPLS) set; and (3) RPLS, in which lattices were selected from the database to best match the homogenized stress field, thereby guiding the failure location away from the central critical zone and producing a failure path opposite to that of BCC (Figure 4b,c). Among these, BCC represents the baseline design as the most widely used fundamental lattice structure in conventional practice; UML serves to test the effect of global strengthening alone; and RPLS embodies the proposed design strategy emphasizing on‐demand configuration.

For the RPLS design, a layered regulation strategy was adopted to achieve controllable redirection of the failure site: First, overall stress homogenization was realized following the proposed methodology. Subsequently, the lattices in the predesignated failure region were replaced with lattices one grade lower in load‐bearing capacity (10 MPa), while the two adjacent layers (along the loading direction) were substituted with stiffer lattices (Figure 4d). This design created local high‐stiffness constraints that forced the weakened middle layer to undergo larger local deformation and to yield and fail first. Through this “clamp–weaken” combination, cracks were induced to initiate and propagate preferentially within the target region, while the critical functional zone was effectively protected by the surrounding reinforcement.

**FIGURE 5:**
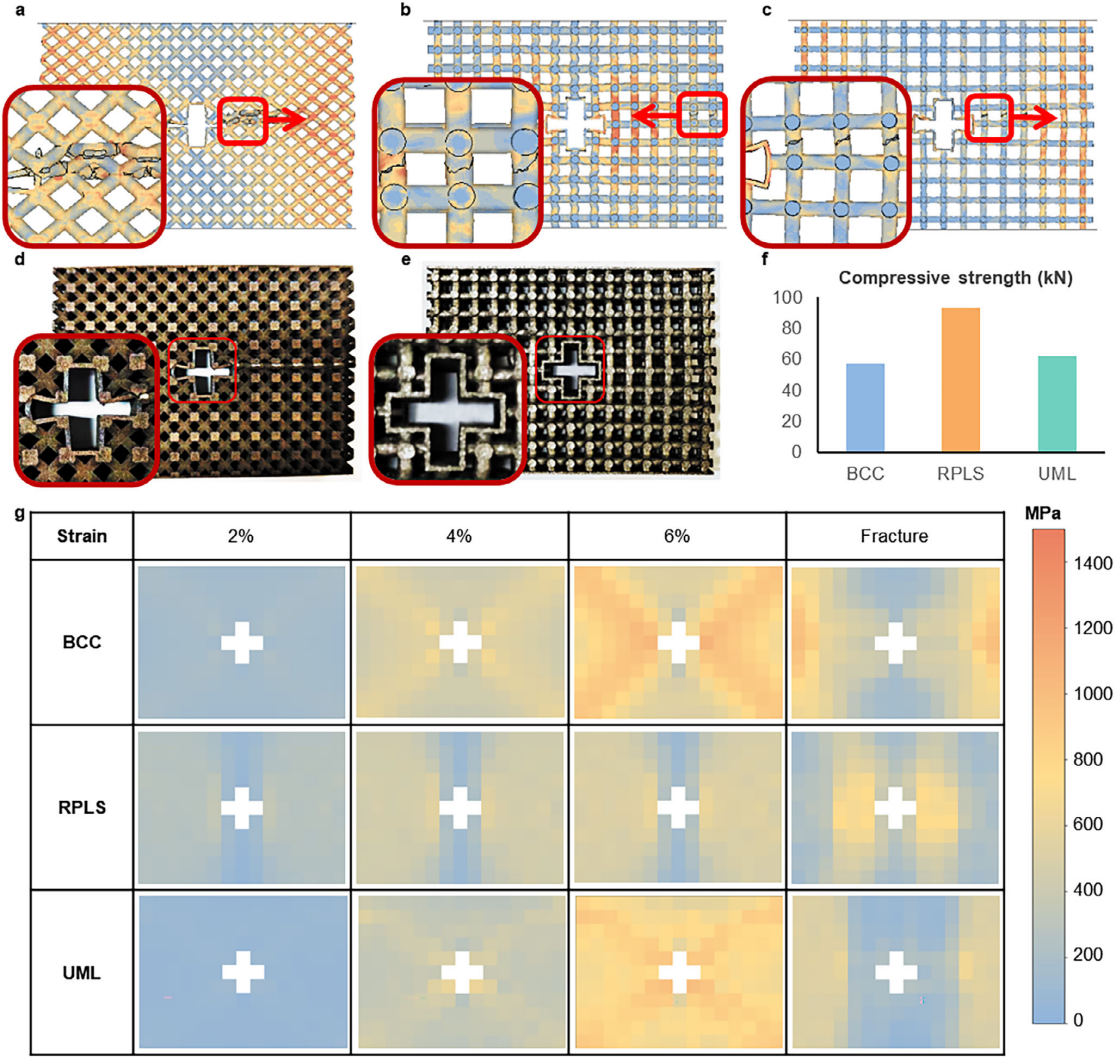
Failure modes and performance of three lattice structures under compressive loading. Red arrows indicate the initial fracture sites and crack propagation paths. (a) BCC structure, with failure initiating in the central critical region and propagating outward. (b) RPLS structure, with significantly reduced stress concentration and failure occurring away from the critical load‐bearing zone. (c) UML structure, with failure initiating in the central critical region and propagating outward. Deformation of the central region after compressive failure in the (d) BCC and (e) RPLS structures. RPLS shows less pronounced deformation than the uniform lattice structure. (f) Comparison of compressive strength: The load‐bearing capacity of RPLS is markedly higher than those of BCC and UML. (g) Comparison of homogenized Mises stress distributions for BCC, RPLS, and UML structures at different strain stages.

Quantitative comparisons showed the advantages of regional regulation (Figure 4f and Table S6). The maximum load capacity of RPLS reached 92.9 kN. This is about 62% higher than uniform BCC (57.2 kN) and 49% higher than UML (62.4 kN). Simulations and compression tests showed differences in failure modes among the three strategies (Figure 5). Both uniform BCC and UML exhibited a central failure mode. Cracks initiated at the cavity edge and rapidly propagated into the core functional zone (Figure 5a,c), ultimately causing premature failure of the critical region. But for RPLS, cracks first appeared at two distal edge regions far from the cavity and then gradually advanced toward the center (Figure 5b), effectively avoiding direct damage to the sensitive zone. RPLS employs multiple lattice configurations and adjacent regions may have different strut diameters. So a graded diameter transition is introduced at the interfacial connections to avoid geometric discontinuities (Figure 3c), while the positions of common nodes are kept identical to ensure connection continuity. Consequently, the interface does not inherently constitute a weak zone. Interface failure depends on the local stiffness contrast and the related load redistribution in the interfacial neighborhood.

In the present case, relatively high‐stiffness regions were intentionally arranged on both sides of the designated interface, creating a pronounced stiffness contrast with the interfacial neighborhood (geometrically manifested as an increase in strut diameter). During loading, this contrast induces load redistribution and pronounced local concentration near the interface, which can be directly observed from the field contours in the interfacial region (Figure 4d), where the regions of high response coincide with the initiation sites of failure. As a complementary observation, scanning electron microscopy (SEM) images of compression fracture surfaces from lattice specimens with different strut diameters are provided in the Supporting Information (Figure S6) to illustrate the evolution of micro‐void coalescence and damage localization with strut scale. These observations do not represent in situ interfacial characterization; rather, they provide a microstructural background for understanding local damage evolution in the interfacial neighborhood under stiffness mismatch. Taken together, the local field evidence and microstructural observations reasonably explain why, under the present design, the designated interfacial neighborhood preferentially becomes the site of damage initiation and failure, thereby enabling controlled failure‐path guidance.

Because the failure location and path in RPLS were designed to be opposite to those of BCC, and because the physical experiments primarily aimed to validate simulation accuracy and fracture control strategies, only BCC and RPLS were compared. Corresponding post‐loading morphologies confirmed this protective effect, with the central region of RPLS showing significantly less deformation than that of BCC (Figure 5b,e).

Under the same relative density (material usage) constraint, the ultimate load‐bearing capacity in RPLS was markedly higher than that in UML, demonstrating that “globally strongest units” do not equate to the “globally optimal design.” In UML, uniform filling with the strongest lattice type produced stress concentration in the critical region during the early stages of loading, resembling the stress distribution state of a solid component. Figure 5g compares the homogenized Mises stress distributions of BCC, RPLS, and UML at different strain levels. Both BCC and UML consistently exhibited pronounced stress concentration, with the core region maintaining the highest stress level throughout loading and ultimately failing first. In contrast, RPLS effectively homogenized stress across regions: In the early stages, the differentiated arrangement diluted peak stress in the core region, yielding a more balanced stress distribution with significantly lower and more uniform overall Mises stress. During subsequent yielding, the outer regions first entered plastic deformation and absorbed energy, while the core region retained elasticity for a longer duration. At final failure, the core region of RPLS still maintained a low stress level, with fracture initiating in the predesigned weakened layers away from the cavity. By adjusting local compliance and stiffness distribution, RPLS enabled a more balanced distribution of energy, outperforming UML in terms of load capacity and realizing coordinated optimization of global performance and local protection. This mechanism explains its enhanced load capacity and shows the unique advantage of the regionally programmable strategy in proactively transferring failure risk.

To show the advantages of the proposed regionally programmable lattice design, RPLS was compared with structures designed by common methods, such as topology optimization and graded‐density lattices, under identical constraints of material usage and manufacturability (Figure S7).

Although topology optimization can efficiently generates rib‐like structures optimized for static stiffness or strength, it inherently sacrifices the continuous, fillable porous region surrounding the protected cavity. This porous zone is critical for multifunctional integration, such as thermal management, acoustic/electromagnetic absorption, or fluid‐related functionalities. In contrast, RPLS achieves targeted mechanical performance while preserving a fully connected lattice architecture, thereby enabling the incorporation of additional functionalities without compromising the primary goal of cavity protection.

Graded‐density lattices are a simpler option and can partially align density variations with the stress field. However, in this specific case, such an approach conflicts with the design principle of maintaining constant relative density and fixed unit‐cell centroid positions. Moreover, its design freedom is fundamentally limited by reliance on a single lattice type, which restricts the ability to realize coordinated multifunctional performance.

These comparisons show that, in engineering scenarios demanding concurrent fulfillment of high load capacity, localized protection, multifunctional porous integration, and manufacturability constraints, RPLS offers a distinct and significant advantage.

This case shows the methodological value of genome‐driven, regionally programmable design for functional protection. It transcends the traditional path of filling with the strongest material and achieves controllable redirection of fracture locations through precise spatial configuration. More importantly, this approach offers a new design paradigm for aerospace protective structures, biomedical implants, and energy absorption devices, and it can be extended to multiphysics responses including impact, thermal management, and fatigue life. Supported by the database, component design is elevated from merely “enhancing local strength” to global risk management, showing the foresight and engineering potential of the Lattice Genome framework for complex functional components.

## 3 Discussion

The Lattice Genome framework is inspired by the data‐driven idea of the Materials Genome Initiative. It establishes a systematic mapping from geometric genotypes to performance phenotypes. In this framework, we propose and validate a genome architecture and a component‐level programmable design strategy tailored for additively manufactured lattices. By constructing a high‐throughput simulation workflow and performance database, we achieved systematic mappings from geometric parameters to mechanical properties and further validated the framework in two representative scenarios: (1) global regulation of lattice unit types enabled stress homogenization under constant density constraints, demonstrating the value of the genome database in expanding design freedom; and (2) regionally programmable filling effectively redirected failure paths and protected critical functional zones, underscoring its engineering potential at the component level.

Traditional approaches such as topology optimization and graded‐density lattices remain highly effective for many design tasks. However, when component design shifts from improving single performance to controlling many goals at the same time, these conventional methods often encounter bottlenecks in both efficiency and practical realizability.

Take topology optimization as an example. It can handle multiple objectives through weighted formulations or constraints, but in practice, it typically requires extensive parameter sweeps over weights or constraint thresholds to approximate the Pareto front, followed by repeated solves for each candidate solution. Moreover, the resulting continuous density fields must then be converted into manufacturable lattice structures, and this step adds more difficulty. Consequently, both computational and post‐processing costs scale dramatically with the number of objectives and the geometric scale of the component.

In contrast, graded‐density lattice approaches operate within a fixed topological framework, modulating local density or strut diameter to achieve gradual property transitions. While conceptually simple, their design freedom is inherently limited. It is hard for this method to keep a continuous functional porous region and at the same time program failure modes or load paths across different regions.

The lattice genome framework proposed in this work addresses these challenges by front‐loading and amortizing the computationally expensive exploration phase into the construction of a unit‐cell genome database via an automated high‐throughput pipeline. This significantly improves efficiency and reduces marginal costs. During component design, multi‐objective control is achieved through regional retrieval and assembly from the database. This removes the need for repeated global iterative optimization for every new component or objective combination.

As a result, when confronted with complex, multi‐objective design requirements, our approach demonstrates clear advantages in design efficiency, reusability, and engineering deployability. It provides a scalable pathway toward intelligent, function‐driven lattice design at the component scale.

These findings show that the genome framework is not merely a static repository of performance data, but a new paradigm driving programmable design and structural optimization. It transforms lattice design from “local empirical optimization” to “global genome‐driven design,” empowering engineers to achieve coordinated function–performance regulation across scales. More importantly, this study reveals a unified pathway for integrating global performance optimization with localized protection, providing a methodological foundation for developing complex functional components with high damage tolerance.

Although regionally programmable lattice structures demonstrate clear advantages in component‐scale stress regulation and failure‐path guidance, the achievable tuning amplitude and spatial resolution are jointly constrained by the response range of the selected lattice families, inter‐regional coupling, and manufacturing limitations. With respect to inter‐regional stress contrast, the upper bound of the proposed method is primarily determined by the range of achievable regional mechanical responses that can be covered by the available lattice configurations under a given material system and process window. In Case 1, a target inter‐regional stress span of approximately 70 MPa was prescribed and was achieved with good agreement. Meanwhile, when the target contrast between adjacent regions becomes excessively large, load redistribution across regions is intensified, reducing the independent controllability of local targets. This effect manifests as deviations of the actual stress level in low‐stress regions from their prescribed values (Figure 3g), indicating that inter‐regional coupling constitutes an effective engineering boundary. For the minimum effective region size, Case 1 demonstrates that unit‐cell level partitioning can still achieve effective regulation (Figure 3f), although the regulation efficacy is similarly influenced by the contrast between neighboring regions and the strength of their coupling.

With respect to the applicability of the proposed method across different machines, processes, and materials, it should be noted that geometric deviations and size‐dependent degradation of mechanical performance are highly sensitive to printing equipment, process parameters, and material characteristics, and they exhibit complex nonlinear behaviors. Accordingly, this study does not explicitly couple the device type or specific process parameters as input variables in a strongly parameterized model. Instead, the combined effects of the device–process–material system on the as‐built outcome are incorporated into two data‐driven correction models: a geometric correction model that characterizes manufacturing‐induced geometric deviations, and a mechanical compensation model that equivalently represents stiffness and strength degradation caused by surface roughness and sub‐scale defects. Both models are embedded within the high‐throughput simulation workflow to enable automated correction of geometric and mechanical deviations for different lattice architectures. Consequently, when changing the printing device, adjusting process parameters, or using different materials (thereby altering the as‐built quality and surface morphology), one can achieve adaptation to new manufacturing conditions by re‐fitting and re‐calibrating these two models using a limited set of new reference samples, without modifying any other components of the overall workflow. This design ensures the scalability and engineering applicability of the proposed method across different devices and process conditions.

Nevertheless, although the proposed Lattice Genome framework demonstrates strong applicability and engineering potential in high‐throughput simulations, database construction, and regionally programmable design, it still has certain limitations. Constrained by time and cost, the current database is primarily built on the performance data of Ti‐6Al‐4 V and Al–Mg–Sc–Zr, without systematic extension to a broader range of material systems. Based on the re‐calibration strategy across different devices, processes, and materials provided in the Supporting Information, ongoing efforts are underway to extend the proposed framework to lattice structures fabricated from a broader range of alloy systems. Second, the coupled effects of residual stress and microstructure in components have not yet been incorporated, which may influence predictive accuracy under extreme service conditions. Finally, while this study validates component‐level programmability mainly under quasi‐static compression, extending the framework to fatigue, impact, and multiphysics service environments will require dedicated damage/strain‐rate modeling and corresponding experimental benchmarking, which we leave for future work.

Internal powder removal also remains a key constraint for the practical engineering application of complex regionally variable‐density lattice structures. The present study primarily focuses on component‐scale regionally programmable mechanical regulation and does not conduct quantitative experiments or powder‐flow simulations specifically targeting the depowdering process itself. To mitigate depowdering risks, manufacturability constraints were introduced at an early stage during the construction of the unit‐cell database, thereby preventing unit cells with enclosed or excessively narrow internal channels from entering the candidate set. In addition, manufacturing‐related attributes were used as screening criteria during component‐level lattice filling. Nevertheless, manufacturability constraints alone are insufficient to fully address depowdering challenges in complex components, as the actual powder‐removal capability also depends strongly on component scale, internal connectivity, and post‐processing procedures. For more demanding engineering applications, depowdering feasibility will be treated as an independent constraint and co‐optimized alongside mechanical objectives in future work. This aims to further improve the applicability of regionally programmable lattice structures.

Future research will further extend the controllability of regionally programmable lattices from static load performance to more complex functional demands, such as fatigue life, impact response, thermal management, and multiphysics coupling. In parallel, systematic evaluation of defect sensitivity and material anisotropy under realistic additive manufacturing conditions is required, calibrated through experimental validation and multiscale simulations. The ultimate goal is to embed programmable lattices into an integrated design–simulation–manufacturing workflow, thereby establishing a complete Lattice Genome database and algorithmic ecosystem for efficient, adaptive design across performances and scenarios, accelerating the engineering translation of complex functional structures. With the continuous expansion of the database and refinement of algorithmic tools, this framework is expected to incorporate predictive capabilities for fatigue, impact, and multiphysics conditions, forming a closed loop through experimental validation and process calibration. In this way, the Lattice Genome framework will not only deepen fundamental understanding of structure–property mappings, but also foster a new paradigm of lightweight, reliable, and multifunctional structural design in additive manufacturing practice.

## 4 Methods

The methodology of this study is built upon the overarching framework of the Lattice Genome for additive manufacturing. First, a high‐throughput finite element simulation approach was established, and the resulting lattice data were used to achieve joint regulation of multiple objectives, including relative density, stress distribution, and failure location. This framework can allow designers to alter the stress distribution state of components under load while satisfying a given mass constraint. It also allows the failure initiation site toward sacrificial zones, which improves the damage tolerance of the component.

### 4.1 Materials and Equipment

Notably, the proposed Lattice Genome framework for additive manufacturing does not depend on a specific material and can be extended to various material systems. This study was aimed at exploring the lattice performance of two commonly used metallic additive manufacturing materials: Ti–6Al–4V and Al–Mg–Sc–Zr alloys. Ti–6Al–4V, owing to its high specific strength, corrosion resistance, and thermal stability, has been widely applied in aerospace applications [53]. Its high specific strength makes its mechanical performance particularly sensitive to geometric design, positioning it as an ideal material for validating programmable lattice design and performance regulation strategies.

Considering these aspects, Ti–6Al–4V alloy powder was selected as the raw material for additive manufacturing. The powder particle size ranged from 15 µm to 53 µm. Specimens were fabricated using a BLT‐S400 selective laser melting system (Bright Laser Technologies, Xi'an, Shaanxi, China). The process parameters corresponded to the optimized parameter set from the equipment's Ti–6Al–4V process package (Table S1). Quasi‐static compression tests of Ti–6Al–4V were performed at room temperature (20°C–25°C).

### 4.2 Lattice Structure Modeling and Mechanical Simulation

As the data foundation of the Lattice Genome framework, ensuring simulation accuracy is essential for subsequent database construction and performance mapping. The primary sources of the discrepancy between simulations and experiments are geometric inconsistencies induced by the staircase effect and the degradation of mechanical performance arising from size‐dependent effects. During additive manufacturing, the inclination angle of lattice struts significantly influences the actual formed diameter, leading to discrepancies between simulation and experimental results. In many previous studies, finite element simulations were performed on idealized models. However, lattice structures are mesoscale architectures whose characteristic lengths fall within the millimeter to sub‐millimeter range, where the stair‐stepping effect of PBF‐L is particularly pronounced [54]. Consequently, the fabricated parts exhibit geometric deviations depending on the strut angle [55], with horizontal struts forming larger diameters than vertical ones [56]. Recent studies across various alloy systems have consistently shown that when characteristic dimensions enter the millimeter scale or below, the effective mechanical properties of specimens decrease markedly with reducing size [43, 44, 45], and that this apparent size effect can be substantially mitigated or even eliminated through appropriate adjustment of process parameters [57]. Such geometric deviations and mechanical performance degradation constitute the primary sources of inconsistency between predicted and measured results, as variations in strut diameter are closely associated with changes in the stress–strain response [58]. To improve the accuracy of finite element simulations, explicitly incorporating process‐induced geometric deviations into the 3D model has proven effective [59].

To account for manufacturing deviations, this study establishes a mapping model between the designed strut diameter and the effective load‐bearing diameter for geometric correction. The model adopts a monotonicity‐constrained Generalized Additive Model (GAM) to capture the stable quasi‐monotonic trend of the effective diameter with respect to build inclination angle and designed strut diameter within the investigated parameter range. GAM is selected because it preserves predictive accuracy while offering strong physical consistency and interpretability, and it avoids the risk of overfitting associated with complex black‐box models under limited sample sizes. The model inputs include the build inclination angle and the designed strut diameter, and the output is the effective diameter characterizing the actual load‐bearing cross‐section.

The effective diameter is extracted from CT‐reconstructed geometries, as described in Methods S1. It should be emphasized that this model is exclusively used for geometric correction (explicitly introducing manufacturing deviations into the finite element geometry) and is not intended for mechanical property prediction. Model training details are provided in MethodsS2. This model was directly embedded into the 3D modeling workflow, enabling finite element simulations of lattice structures to realistically capture process‐induced geometric deviations (Figure S3).

The 3D lattice models were generated using CAD software and automatically exported in both STL and STEP formats. The STL models were used for printing lattice structures on the BLT‐S400 system, whereas STEP models were employed for finite element analysis (FEA). The simulation environment was established in ABAQUS 2024 (Dassault Systèmes, Vélizy‐Villacoublay, France) under the same conditions as the mechanical experiments, using the ABAQUS/Explicit solver to capture failure behaviors highly consistent with experimental results [60].

In this analysis, the lattice strut diameters ranged from 0.5 mm to 1.5 mm. Studies have shown that within this range, strut size exerts negligible influence on the microstructure, and post‐processing does not significantly affect the fundamental mechanical response during compression [61]. Moreover, in PBF‐L‐fabricated titanium alloys, the grain size is at least one order of magnitude smaller than the strut diameter, and no preferential crystallographic orientation has been observed [62]. Therefore, heterogeneity of material properties across micro‐ and mesoscales was not considered in the numerical analyses.

To account for the degradation of mechanical performance induced by size effects, a mechanical compensation procedure is further introduced to reduce the systematic discrepancy between finite element simulations and experimental measurements. Previous studies have consistently shown [43, 44, 45] that, when characteristic dimensions enter the millimeter scale or below, the degradation of mechanical performance is primarily driven by surface roughness effects (namely, the reduction of the effective load‐bearing cross‐section and the associated stress concentration) and follows a stable monotonic deterioration trend with decreasing strut size. Based on this well‐established physical mechanism, the present study treats the compensation process as a physically consistent calibration problem: using the effective load‐bearing diameter *d*_eff_ obtained from CT reconstruction as the sole independent variable, a 1D mapping between diameter and performance degradation (or equivalently, effective material parameters) is constructed to calibrate the effective constitutive parameters in the finite element model, including the elastic modulus, yield strength, plastic hardening parameters, and damage parameters.

Given the low dimensionality of this mapping, the limited number of data points, and the presence of a clear monotonic physical prior, low‐order cubic polynomial interpolation is adopted to represent these relationships, rather than introducing more complex machine‐learning models, in order to enhance interpretability and reduce the risk of overfitting. It should be noted that this study does not independently adjust process parameters for different characteristic sizes; instead, defect effects introduced under the prescribed processing conditions are explicitly incorporated into the compensation workflow through CT‐based geometric reconstruction and experimental benchmarking. As a result, manufacturing‐induced defects are not idealized or neglected, but are embedded into the simulation‐correction workflow in the form of calibrated inputs and equivalent parameters.

After the geometric model and constitutive parameters are independently calibrated based on experimental data, they are coupled within the simulation workflow and revalidated by comparing the simulation results obtained using the geometrically corrected model and the calibrated equivalent material parameters with experimental measurements. In this study, the simulated and experimental results show good agreement, and the relative deviation is no more than 5%. This indicates that the geometric correction model and the mechanical compensation model achieve sufficient accuracy and can serve as a stable foundation for high‐throughput computations.

### 4.3 High‐Throughput Simulation Platform

To efficiently obtain performance data across a large design space, we developed a fully automated high‐throughput simulation platform. Designed to rapidly evaluate lattice performance, this platform incorporates a fully automated workflow integrating 3D modeling, FEA, and data processing. The performance data acquired through high‐throughput simulations are written into the lattice structure database (**Methods** **S5**), serving as the data source for component design. In the Lattice Genome framework, this strategy enables the exploration of new design spaces through automated modeling, significantly reduces the experimental burden by substituting physical tests with finite element simulations [63, 64], leverages machine learning to accelerate performance prediction [65, 66], and facilitates simultaneous analysis of nonlinear structure–property relationships in lattices [67]. Collectively, these steps establish a data foundation for multi‐objective lattice optimization and differentiated filling under specific performance targets or localized mechanical response requirements.

To acquire a large volume of lattice data for precise performance regulation and subsequent machine learning, lattice structures were generated using a computer‐based random approach. First, lattices were categorized according to the numbers of boundary nodes (6, 8, 10, or 12) and internal nodes (1 to 8). To ensure connectivity, boundary nodes were fixed (Figure S5a), while internal nodes and strut positions were randomly distributed (Figure S5b). To ensure manufacturability, geometric constraints are imposed at the unit‐cell generation stage, including limits on overhang angles, maximum unsupported spans, and minimum feature sizes. As a result, all unit cells stored in the database are directly printable within the prescribed manufacturing capabilities and can be reused in the automated component‐filling process without introducing locally unprintable geometries. Detailed design constraints, based on truss characteristics and additive manufacturing considerations, are provided in Methods S3.

Modeling used Open CASCADE Technology (Open CASCADE S.A.S., Nanterre, France), and a Python program was developed for 3D lattice generation. Specifically, the node coordinates of each lattice were stored in a TXT file, and connectivity (node indices to be joined) was recorded in another TXT file to define strut positions. Spheres were placed at each node location to represent joints, and cylinders were created between connected nodes to represent struts, forming a complete unit cell. The relative density of the unit cell was calculated. Strut diameters were iteratively adjusted and the cell was regenerated until the desired relative density was reached. Last, arrays of unit cells were assembled to produce a complete lattice structure (Figure S5c).

FEA was used to predict the mechanical properties and deformation behavior of lattice structures under compressive loading. Based on secondary development in ABAQUS, the simulation process was implemented in Python scripts. The model import path, material parameters, and loading parameters was defined as variables to allow easy modifications and batch simulations. After each FEA run, Python scripts were used to extract reaction force and displacement data from the ODB result files at specified nodes. These data were processed and exported as CSV files to generate force–displacement and stress–strain datasets.

The unit cell size used for the simulations was 5 mm × 5 mm × 5 mm, arranged in a 4 × 4 × 4 configuration. According to related studies [68, 69], this arrangement incorporates a complete configuration for stress transfer between adjacent lattice cells, thereby yielding acceptable boundary effects while maintaining high computational efficiency.

To validate the high‐throughput simulation results, different lattice structures were selected for comparison with experimental observations. The relative density of the lattices ranged from 0% to 100%. To cover the full density spectrum while balancing efficiency, four representative relative densities—20%, 40%, 60%, and 80%—were chosen for compression simulations. Corresponding lattice specimens were fabricated and subjected to quasi‐static compression tests, enabling comparison between simulations and experiments to verify whether the high‐throughput simulation program could accurately capture the mechanical performance of lattice structures.

Specimens were placed between movable and fixed platens and compressed until failure using the E45.305 universal testing machine (MTS System Corporation, Eden Prairie, Minnesota, USA) at a nominal strain rate of 10^−3^. The nominal stress was calculated as the ratio of the reaction force to the initial contact area of the specimen, and the nominal strain was defined as the compression displacement divided by the initial specimen height.

### 4.4 Machine‐Learning Prediction of Lattice Compressive Performance

The machine learning module serves as the predictor within the Lattice Genome framework. It uses large amounts of data from the database to establish mappings between structural genotypes (geometric and topological parameters) and phenotypes (mechanical properties), thereby enabling rapid performance inference. Given the nearly infinite number of theoretically possible unit‐cell configurations, using machine learning to predict the performance of additional structures accelerates the progress of the Lattice Genome initiative.

According to the computational results (Figure 2b), lattices with six boundary nodes and one internal node exhibited the widest range of elastic modulus and compressive strength. Therefore, to ensure adequate regulatory space for subsequent joint control of relative density and stress distribution in components, machine‐learning predictions were obtained over this dataset. This necessitated a sufficiently large dataset; thus, high‐throughput simulations were used to generate a dataset that covered the complete design space and satisfied training demands.

We constructed two supervised learning models to predict the elastic modulus and compressive strength, respectively. Considering the presence of manufacturing noise and small‐sample subregions in high‐throughput data, and with an emphasis on engineering deployability and predictive stability, a stacking ensemble framework was adopted. Specifically, Random Forest and Linear Regression were used as base learners to capture nonlinear high‐order interactions and global linear trends, respectively, while Ridge Regression served as the meta‐learner to regularize and fuse the base‐learner outputs, thereby mitigating multicollinearity and reducing the risk of overfitting.

The training data were obtained from high‐throughput simulations, with 80% of the samples randomly assigned to the training set and the remaining 20% reserved as an independent test set. The input features consist of low‐dimensional parameters with clear physical meaning (the 3D coordinates of internal nodes), and the outputs are the corresponding elastic modulus or compressive strength. Hyperparameter optimization was conducted on the training set using Optuna, with candidate configurations evaluated via fivefold cross‐validation. The Optuna objective function was defined to maximize the cross‐validated mean *R* ^2^, with a search budget of TRIALS = 100 and early stopping enabled to avoid ineffective searches and overfitting. The model was then retrained on the full training set using the optimal hyperparameters, and the generalization error was finally reported on the independent test set.

In addition, XGBoost and Multi‐Layer Perceptron (MLP) models were trained as comparative baselines using exactly the same dataset and hyperparameter optimization protocol. To further assess model stability under continuous design perturbations, a 1D scan analysis (fixing all other variables at their median values while varying a single variable) was performed to compare the smoothness of predictive responses and the distribution of residuals (Figure S2). Implementation details and comparative results are provided in Methods S4.

### 4.5 Regionally Programmable Lattice Design Method

To achieve component‐level regional performance regulation, we propose a programmable lattice design method driven by multi‐objective fields. The core concept is to map the design of complex components into a process of “gene selection and recombination” within the Lattice Genome framework: Functional or mechanical requirements in different regions are translated into phenotypic constraints, while the geometric parameters and performance curves of lattice unit cells stored in the database serve as candidate genotype–phenotype pairs. Under these phenotypic constraints, the most suitable genotype–phenotype pairs (i.e., unit cells and their corresponding performance) are retrieved, rearranged, and recombined at the regional level, enabling coordinated regulation of both global and local performance.

Specifically, in this analysis, the component was divided into spatial regions according to a predefined unit size (5 mm × 5 mm × 5 mm), and FEA of the solid component was performed under given boundary conditions to obtain the global stress distribution. The stress in each region was homogenized and rescaled to fit within the achievable stress range of lattice unit cells. This produced the phenotypic requirements that reflect local loading characteristics. Based on these requirements, the most similar lattice unit type and its stress–strain curve were retrieved from the performance database. The predictor was applied to conduct a grid search in the neighborhood of the unit's geometric parameters to refine the matching. The lattice most closely aligned with the phenotypic requirements was selected as the candidate gene for the region and filled into the corresponding spatial position, resulting in a differentiated distribution and a programmable lattice configuration. Unlike conventional approaches that require manual selection of lattice types, the 3D modeling of components is a field‐driven automated process, which eliminates the need for labor‐intensive model construction and enables rapid generation of complex geometries.

The generated structural models were revalidated in the finite element environment to assess the effects of regional filling on global performance and local protection. If stress remained concentrated in the critical functional region or deviated substantially from the target distribution, iterative optimization was performed by adjusting the regional partitioning or reselecting genotypes from the database. In this study, iteration was considered complete when the failure site was redirected away from the critical region or when the deviation between the stress distribution and target was less than 10%. This workflow established a closed loop from stress‐field analysis to database use and made regional design a natural extension of the Lattice Genome framework. In other words, through spatial selection and recombination of genes, components achieved coordinated optimization of both global and local performance.

Overall, this methodology constitutes not merely a set of isolated experiments or simulations but a comprehensive Lattice Genome platform: High‐throughput simulations are employed to explore lattice phenotypes, databases and machine learning are used to establish structure–property mappings, and regionally programmable design enables gene recombination and phenotype regulation. This framework provides a universal foundation for future extensions to multiphysics performance optimization and cross‐material applications.

## Author Contributions

H. Deng conceived the idea, designed the study, performed experiments and database construction, and wrote the manuscript. Y. Zhao provided overall guidance and assisted in data analysis and manuscript preparation. M. Cao assisted with specimen fabrication, CT scanning, and geometric deviation analysis. H. Yang provided guidance on additive manufacturing processes and experimental design. P. Wang contributed to finite element simulations. H. Wang provided machine learning model development. H. Wang assisted with data post‐processing and figure preparation. A. Chiba provided critical discussions on methodology and implications. X. Lin supervised the project and revised the manuscript. All authors discussed the results and approved the final manuscript.

## Funding

National Key R&D Program of China (Grant No. 2023YFB3712000 and Grant No. 2024YFE0105700). Natural Science Basic Research Program of Shaanxi (Program No. 2025SYS‐SYSZD‐038). Fundamental Research Funds for the Central Universities (Grant No. G2024KY0616). Shaanxi Provincial Foundation for Selected Scientific Research Activities of Returned Scholars (Grant No. 2024001). National Natural Science Foundation of China (Grant No. 52441503). National Science and Technology Major Project (Grant No. 2026ZD16011800)

## Conflicts of Interest

The authors declare no conflicts of interest.

## Supporting information




**Supporting File 1**: advs74357‐sup‐0001‐SuppMat.docx.

**Supporting File 2**: advs74357‐sup‐0002‐SuppMatSupplementary_Video_1.mp4.

**Supporting File 3**: advs74357‐sup‐0003‐SuppMatSupplementary_Video_2.mp4.

**Supporting File 4**: advs74357‐sup‐0004‐SuppMatSupplementary_Video_3.mp4.

**Supporting File 5**: advs74357‐sup‐0005‐SuppMatSupplementary_Video_4.mp4.

## Data Availability

The lattice performance database generated and analyzed in this study is not publicly available at this stage owing to ongoing extensions of the Lattice Genome framework, but it can be obtained from the corresponding author upon reasonable request.
