# Supplementary material for: Lattice Genome Framework for Regionally Tailored Component‐Level Multi‐Objective Design in Additive Manufacturing
Source: Adv Sci (Weinh). 2026 Feb 15;13(22):e22126. doi: 10.1002/advs.202522126 (PMC13088266; doi:10.1002/advs.202522126)
Supplement: Supplementary file 1 — Supporting File 1: advs74357‐sup‐0001‐SuppMat.docx. [file ADVS-13-e22126-s001.docx]

Supporting Information

**Lattice Genome Framework for Regionally Tailored Component-Level Multi-Objective Design in Additive Manufacturing**

### *Haoyuan Deng^a^, Yufan Zhao^*a, b^, Mingyang Cao^a^, Haiou Yang^a,b^, Pan Wang^c^, Hongze Wang^d^, Hao Wang^e^, Akihiko Chiba^f^ and Xin Lin^*a, b^*

### ^a^ State Key Laboratory of Solidification Processing, Northwestern Polytechnical University, Xi’an 710072, PR China

### ^b^ Key Laboratory of Metal High Performance Additive Manufacturing and Innovative Design, MIIT China, Northwestern Polytechnical University, Xi’an 710072 PR China

### ^c^ Singapore Institute of Manufacturing Technology (SIMTech), Agency for Science, Technology and Research (A*STAR), 5 Cleantech Loop 636732, Republic of Singapore

^d^ School of Materials Science & Engineering, Shanghai Jiao Tong University, Shanghai 200240, PR China

### ^e^ Co-Creation Institute for Advanced Materials, Shimane University, Matsue, Shimane 690-8504, Japan

### ^f^ New Industry Creation Hatchery Center (NICHe), Tohoku University, Sendai, Miyagi 980-8577, Japan

### ^*^ Corresponding authors:

### Email address: xlin@nwpu.edu.cn (L. Xin) & zhyf90215@nwpu.edu.cn (Y. Zhao)

# **Supplementary Methods**

### **Methods S1. Characterization of Strut Diameter Deviation**

### To quantify the deviation between the designed and as-built diameters of struts, a semi-circular specimen was designed. The specimen incorporated struts with inclination angles ranging from 0° to 90° at 15° intervals, with two additional support struts included to meet the manufacturability requirements of the PBF-L process (**Figure S3a**). Based on typical strut sizes in lattice structures, specimens with nominal diameters of 0.4, 0.6, 0.8, 1.0, 1.2, 1.6, and 2.0 mm were fabricated.

### The fabricated samples were scanned using industrial computed tomography (CT) to obtain the actual strut diameters. The results were compared against struts of the same inclination and nominal diameter within lattice structures, including assessments of powder adhesion. This confirmed that the struts in the designed calibration specimen exhibited consistent dimensional deviations and adhesion phenomena with those in lattice structures (**Figure S3b**).

### A machine-learning model was trained using strut inclination angle and nominal diameter as input variables, and the measured as-built diameter as the output. The trained model was subsequently embedded into the automated 3D modeling process, thereby generating geometry that accounts for manufacturing-induced deviations (**Figure S3c**). This correction improved the accuracy of subsequent finite element simulations.

### **Methods S2. Generalized Additive Model (GAM) for Diameter–Inclination Compensation**

### To quantify and correct the systematic deviation between the nominal strut diameter and the fabricated diameter under different build inclinations, a generalized additive modeling (GAM) framework was employed.

### The dataset consists of three variables:

### Input 1: The inclination angle θ (0–90°) was used as the predictor. A monotonicity prior (increasing relationship) was enforced in the GAM smooth term for θ to ensure physically consistent trends.

### Input 2: Nominal strut diameter *d*_design_ (mm).

### Output: Measured strut diameter *d*_eff_ (mm).

### To ensure robust performance evaluation and avoid distributional bias, the dataset was divided using an 80 / 20 train–test split.

### Model selection and hyperparameter tuning were conducted exclusively on the training set using 5-fold cross-validation (CV). The test set was held out and used only for final performance evaluation.

### Two key hyperparameters were optimized:

### Smoothing parameter *λ*, controlling the trade-off between fidelity and smoothness.

### Number of spline basis functions *N*_splines_, determining the flexibility of each smooth term.

### A grid search was performed over:

### *λ* ∈ [10^-4^, 10^4^] (logarithmically spaced),

### *N*_splines_ ∈ {10, 15, 20, 25}.

### For each hyperparameter combination, the mean squared error (MSE) averaged over the 5 CV folds was computed. The configuration minimizing the cross-validated MSE was selected as optimal.

### Model performance was quantified using MSE and the coefficient of determination (*R*²). With the optimal configuration, the GAM achieved an MSE of 0.004 mm² and an *R*² of 0.98 on the held-out test set, indicating high fidelity in capturing the inclination-dependent diameter deviation.

### **Methods S3. Design Parameters and Constraints for Automated Lattice Generation**

### The geometric generation of lattice unit cells is governed by the following design parameters and process constraints, ensuring reliable manufacturability of all generated structures.

### Boundary node definition: number and positions of nodes located on lattice edges or faces.

### Internal node definition: number and positions of nodes located inside the unit cell.

### Strut configuration: positions of struts connecting boundary and/or internal nodes.

### Connectivity limitation: the maximum number of struts per node, to avoid excessive stress concentration.

### Process-driven restrictions: maximum allowable strut inclination and void size, ensuring manufacturability under PBF-L process constraints.

### Symmetry requirement: definition of symmetry within the unit cell, which governs anisotropy of mechanical responses.

### Connectivity enforcement: each internal node must connect to at least one boundary node on both the upper and lower halves of the lattice cell, ensuring global stability and manufacturability.

### **Methods S4. Rationale, benchmarking, and stability analysis of the RF–LR–Ridge stacking model**

### To justify the architectural choice of the supervised predictor used in this study, we conducted baseline benchmarking and stability analyses under identical datasets and evaluation protocols, and accordingly selected a robust stacking framework as the primary prediction model. This design prioritizes not only pointwise accuracy but also prediction stability under small input perturbations and manufacturing-related noise, which is critical for downstream optimization and region-wise design decisions. Specifically, RF captures nonlinear interactions and higher-order feature coupling, LR provides an interpretable global linear baseline, and Ridge regularizes the fusion of base-learner outputs, mitigating multicollinearity and reducing overfitting risk in small-sample subregions.

### For benchmarking, we additionally trained two mainstream models, XGBoost and Multi-Layer Perceptron (MLP), using the same input features, identical train/test split, and consistent evaluation metrics (*R*²). Hyperparameters of all models were optimized under the same protocol using Optuna, where the objective function maximizes the mean cross-validated R² (5-fold CV) with 100 trials and early stopping. Overall, the three models exhibit comparable prediction accuracy, with test-set *R*² for compressive strength values of XGBoost: 0.926, MLP: 0.931, and RF–LR–Ridge stacking: 0.923; test-set *R*² for elastic modulus values of XGBoost: 0.911, MLP: 0.923, and RF–LR–Ridge stacking: 0.914.

### To assess model stability beyond aggregate metrics, we performed a sensitivity-based 1D scan analysis (**Figure S2a, b**). In each scan, one variable was swept continuously while the remaining variables were fixed at their median values, and the predicted responses were examined for functional smoothness and error behavior. The XGBoost predictor exhibits more pronounced local step-like fluctuations under continuous input perturbations, suggesting a higher sensitivity to small geometric variations. In contrast, the RF–LR–Ridge stacking model follows a smoother response curve that is more consistent with the expected continuity of the underlying structure–property mapping. The MLP predictions are also relatively smooth but display a noticeable bias in the peak-response regime.

### Importantly, the residual analyses (**Figure S2c, d**) further support the robustness of the stacking strategy. For both elastic modulus and compressive strength, the stacking model shows residual histograms and KDE curves that are more concentrated and centered closer to zero, indicating reduced systematic bias and a more uniform error distribution. By comparison, XGBoost and MLP exhibit broader residual spreads and/or a clearer shift of the residual density away from zero in certain regimes, implying less homogeneous generalization across the sampled range. Such locally irregular responses and non-uniform residual patterns can accumulate and propagate in subsequent optimization or inverse-design iterations. Considering accuracy, stability, and engineering deployability, we therefore adopt the RF–LR–Ridge stacking model as the primary predictor in the proposed workflow.

### **Methods S5. Database Construction and Content Description**

### The database developed in this study is built on MySQL and currently consists of five categories of data tables: a materials table, a geometry table, a nondestructive testing (NDT) table, a mechanical properties table, and a reserved functional properties table.

### Materials table: Records fundamental physical properties (e.g., density) and process-related characteristics such as manufacturing methods, enabling comparison and traceability across different materials (**Table S2**).

### Geometry table: Stores geometric parameters that uniquely define lattice types, including node coordinates and connectivity, or parameterized functional expressions (**Table S3**).

### Nondestructive testing table: Contains information on structural integrity such as porosity and relative density measured from selected lattice specimens (**Table S4**).

### Mechanical properties table: Provides mechanical response data including elastic modulus, strength, and other performance metrics of various lattice types (**Table S5**).

### Functional properties table (reserved): Designed for future extensions, covering multifunctional characteristics such as energy absorption and electromagnetic wave attenuation.

### At present, the database includes two widely used additive manufacturing materials, Ti-6Al-4V and Al–Mg–Sc–Zr, with additional alloys being progressively incorporated. A front-end visualization interface has been developed for the database, and future plans aim to make it publicly accessible, allowing researchers worldwide to share data and contribute their uniquely designed lattice structures. This initiative is expected to accelerate the collaborative advancement of the Lattice Genome framework.

# **Supplementary Figures**

## **Figure S1. Machine learning prediction results.**

###
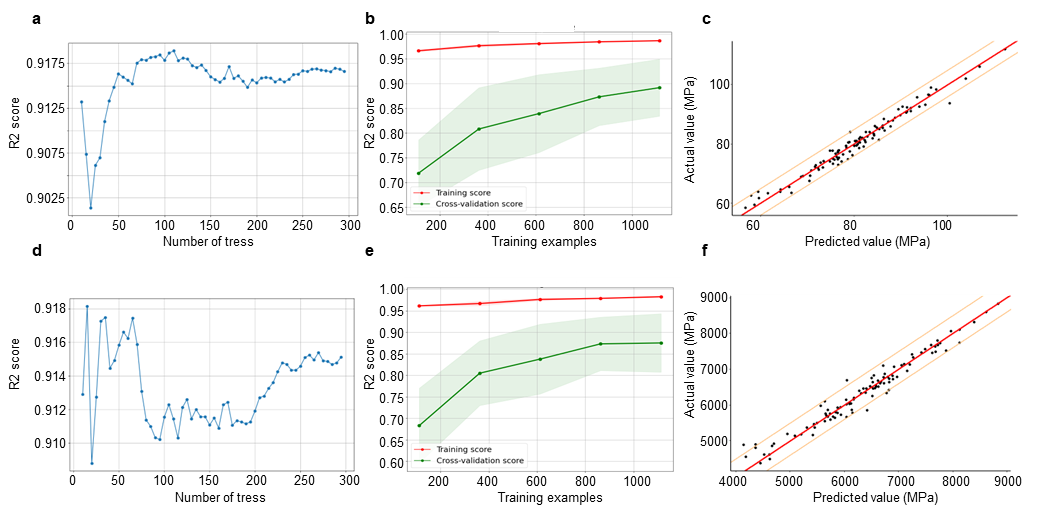


**a** Hyperparameter tuning for the compressive strength prediction model.

**b** Learning curve for compressive strength.

**c** Predicted versus actual values of compressive strength.

**d** Hyperparameter tuning for the elastic modulus prediction model.

**e** Learning curve for elastic modulus.

**f** Predicted versus actual values of elastic modulus.

## **Figure S2. Performance comparison of three surrogate models (XGBoost, MLP, and stacking) for predicting lattice mechanical properties.**

###
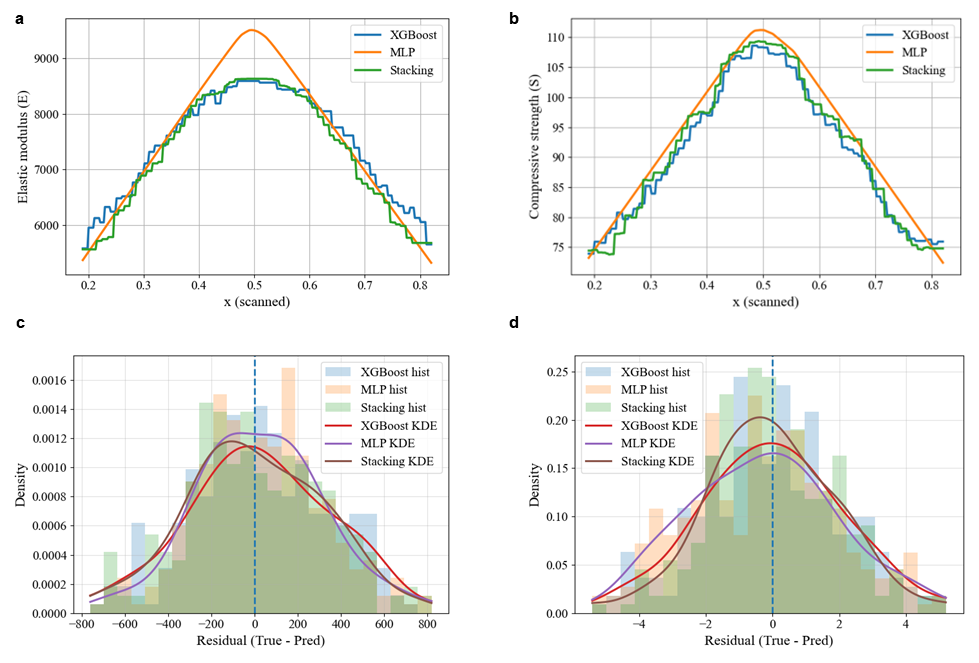


**a, b** One-dimensional scan sensitivity analysis in which one input variable *x* is scanned while all other variables are fixed at their median values, showing the model responses for elastic modulus and compressive strength respectively.

**c, d** Residual distributions (True − Pred) for elastic modulus and compressive strength, presented as histograms with kernel density estimates, where the dashed vertical line marks zero residual.

Overall, the stacking model (RF + LR → Ridge) exhibits comparable pointwise accuracy while providing a smoother response under continuous perturbations and a more uniform residual distribution, supporting its use as the primary predictor in subsequent design iterations.

## **Figure S3. Measurement of strut diameter deviation and modeling workflow incorporating geometric defects.**

###
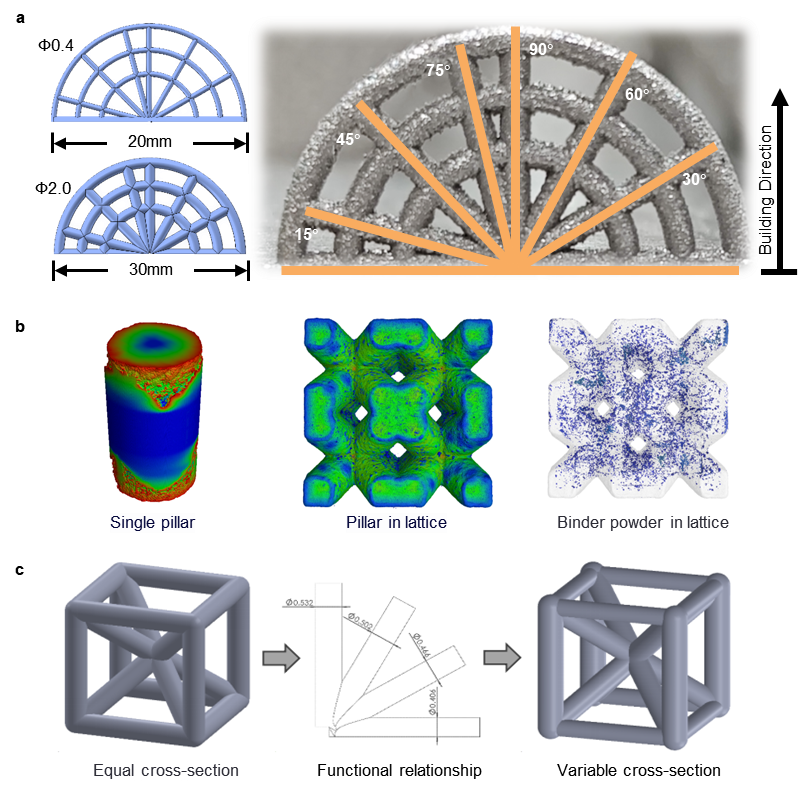


### **a** Designed semi-circular specimen containing struts oriented from 0° to 90° at 15° intervals, with additional supports on both sides to satisfy PBF-L process constraints; strut diameters were set to seven sizes ranging from 0.4 to 2.0 mm.

### **b** Industrial CT scan of the fabricated specimen, used to obtain actual strut diameters and compare diameter deviations and powder adhesion with lattice struts of corresponding angles and diameters.

### **c** Machine learning model trained with strut angle and designed diameter as inputs and actual diameter as output, integrated into the 3D modeling workflow to generate lattice models incorporating process-induced geometric defects, thereby improving the accuracy of finite element simulations.

## **Figure S4. Workflow of the high-throughput simulation correction strategy.**


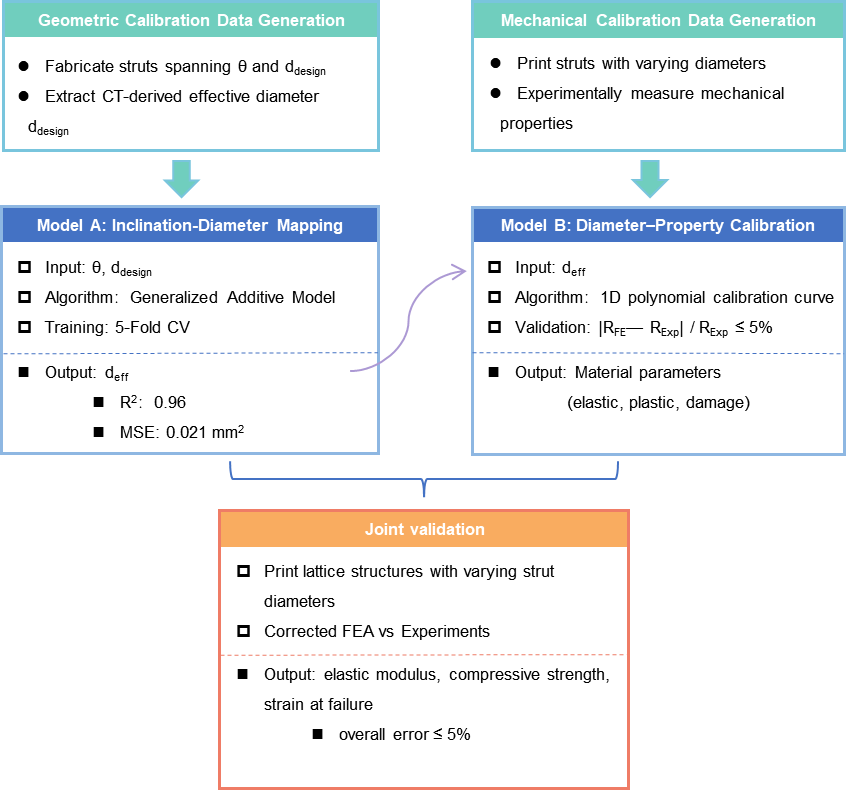


Geometric calibration data are generated by fabricating struts spanning the build inclination angle (*θ*) and the designed diameter (*d*_design_), followed by CT reconstruction to extract the effective load-bearing diameter (*d*_eff_). A supervised mapping model (Model A, generalized additive model; input: *θ* and *d*_design_; output: *d*_eff_) is trained using 5-fold cross-validation to predict geometry-corrected diameters. Mechanical calibration data are obtained from compression tests of struts with varying diameters, and a one-dimensional polynomial calibration curve (Model B; input: *d*_eff_ ) is established to calibrate the constitutive parameters (elastic, plastic, and damage) such that the relative discrepancy between corrected finite element analysis (FEA) and experiments satisfies ∣*R*_FE_ – *R*_Exp_∣ / *R*_Exp_ ≤ 5%. The two models are then jointly validated on lattice specimens with varying strut diameters by comparing corrected FEA predictions (elastic modulus, compressive strength, and strain at failure) against experiments, yielding an overall error below 5% within the investigated design space.

## **Figure S5. Workflow of computer-automated modeling for generating random lattice structures.**


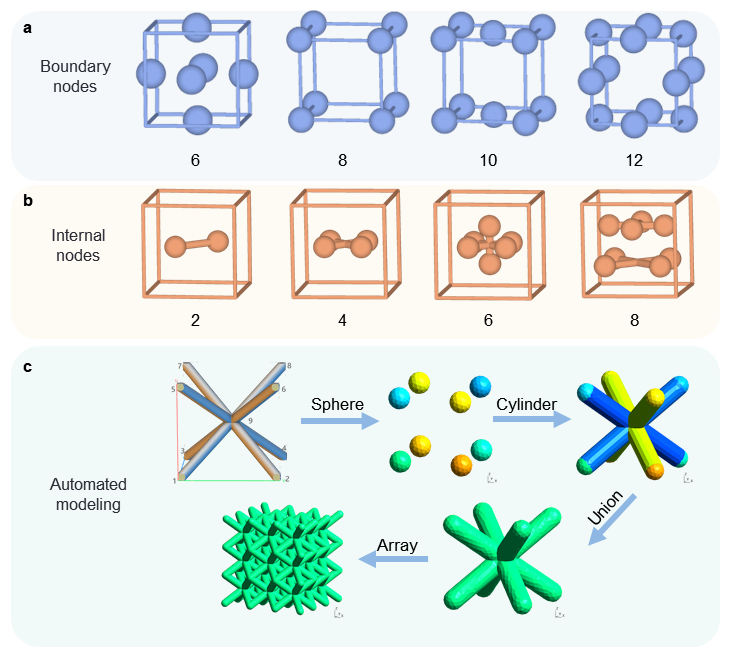


**a** Lattices are first categorized by the number of boundary nodes and internal nodes. The boundary node number is predefined (e.g., 6, 8, 10, 12) and fixed in distribution to ensure connectivity.

**b** The number of internal nodes ranges from 1 to 8, with their positions and connections randomly generated.

**c** Using the Open CASCADE kernel (Matra Datavision, France), a Python-based program performs 3D modeling: node coordinates and connectivity indices are written into text files, and the program generates spheres at the nodes and cylinders between connected nodes to form a complete unit cell. The relative density of the unit cell is then calculated and iteratively updated by adjusting strut diameters until the target density is reached. Finally, unit cells are arrayed to construct the complete lattice structure.

## **Figure S6. SEM images of fracture surfaces from compressed lattice specimens for struts with different diameters.**


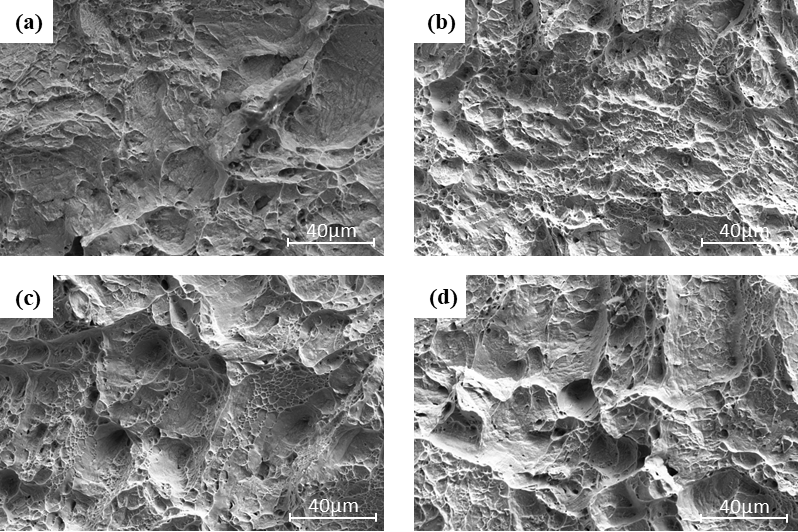


**a** 0.5 mm strut diameter.

**b** 0.8 mm strut diameter.

**c** 1.1 mm strut diameter.

**d** 1.4 mm strut diameter.

All cases predominantly exhibit ductile micro-void nucleation–growth–coalescence features (dimples with tearing ridges). With increasing strut diameter, the dimple morphology becomes more developed and spatially continuous, indicating a diameter-dependent tendency of damage localization and plastic energy dissipation. These observations provide microstructural context for understanding interface-near damage evolution associated with diameter/stiffness mismatch in regionally programmed lattices.

## **Figure S7. Comparison with topology optimization and graded-density lattice structure for the cavity-protection case.**


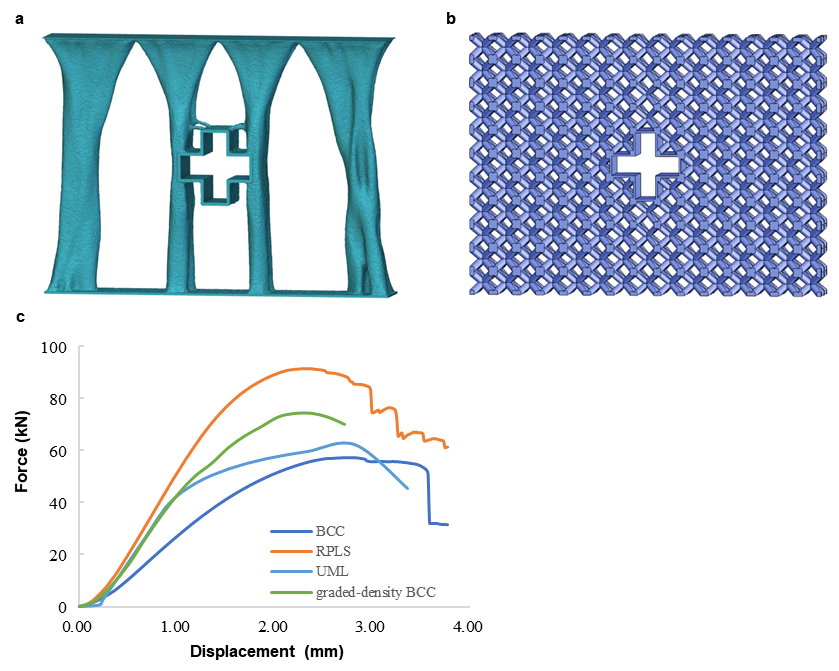


**a** Topology-optimized structure.

**b** Graded-density BCC structure.

**c** Comparison of force–displacement curves for four structures, including BCC (max 57.2 kN), RPLS (max 92.9 kN), UML (max 62.4 kN), Graded-density BCC (max 74.1 kN).

# **Supplementary Tables**

## **Table S1. Additive manufacturing process parameters.**

| **Spot size**  **(μm)** | **Layer thickness**  **(mm)** | **Scanning speed**  **(m/s)** | **Hatch spacing**  **(mm)** | **Laser power**  **(W)** |
| --- | --- | --- | --- | --- |
| 60 | 0.04 | 1250 | 0.08 | 250 |

## **Table S2. Material data table.**

| **Name** | **Type** | **Default** | **Comment** |
| --- | --- | --- | --- |
| MAT_ID | Int(11) | AUTO_INCREMENT | Material ID |
| MAT_NAME | Text | NULL | Material Grade |
| MAT_COMPOSITION | Json | NULL | Chemical Composition |
| MAT_MANUFACTURING | Text | NULL | Preparation Method |
| MAT_PRODUCERS | Text | NULL | Manufacturer |

### The materials table is designed to accommodate the inclusion of new materials in the future, ensuring sufficient flexibility and scalability of the database. It records information such as alloy designation, chemical composition, powder production method, and manufacturer, enabling both traceability and comparative analysis when new materials are added.

## **Table S3. Lattice structure geometric parameters data table.**

| **Name** | **Type** | **Default** | **Comment** |
| --- | --- | --- | --- |
| LAT_ID | Int(11) | AUTO_INCREMENT | Lattice ID |
| LAT_NAME | Text | NULL | Lattice Name |
| LAT_ABB | Text | NULL | Abbreviation |
| LAT_COOR | Json | NULL | Node Coordinates |
| LAT_CONN | Json | NULL | Column Number |

### The geometry table provides a detailed record of all node coordinates and strut connectivity within the lattice structure, ensuring geometric precision and consistency. The node coordinate section specifies the three-dimensional spatial positions of each node, describing the spatial distribution of the lattice. The strut indexing section defines the connectivity between nodes, thereby determining the positions of struts. Together, these geometric parameters fully capture the essential characteristics of the lattice structure and provide a complete data foundation for automated modeling.

## **Table S4. CT nondestructive testing data table.**

| **Name** | **Type** | **Default** | **Comment** |
| --- | --- | --- | --- |
| CT_ID | Int(11) | AUTO_INCREMENT | Data ID |
| CT_LATTICE | Text | NULL | Lattice Name |
| CT_MATERIAL | Int(3) | NULL | Material ID |
| CT_UCS | Json | NULL | Unit Cell Size |
| CT_DEN | Decimal(10,2) | NULL | Relative Density |
| CT_PRO | Float | NULL | Porosity |
| CT_SP | Float | NULL | Surface Powder Adherence Rate |

### The nondestructive testing table contains characterization results obtained without damaging the specimens, including porosity, surface powder adhesion ratio, and average strut diameter. This table provides essential references for analyzing build quality and performance under varying process parameters in future studies.

## **Table S5. Mechanical properties data table.**

| **Name** | **Type** | **Default** | **Comment** |
| --- | --- | --- | --- |
| COM_ID | Int(11) | AUTO_INCREMENT | Data ID |
| COM_LATTICE | Text | NULL | Lattice Name |
| COM_MAT | Int(3) | NULL | Material ID |
| COM_DEN | Decimal(10,2) | NULL | Relative Density |
| COM_MODULUS | Float | NULL | Elastic Modulus |
| COM_STRENGTH | Float | NULL | Compressive Strength |
| COM_UCS | Json | NULL | Unit Cell Size |
| COM_SIZE | Json | NULL | Overall Size |
| COM_RAW | Json | NULL | Raw Data |
| COM_SURFACE | Float | NULL | Cross-sectional Area |
| COM_LENGTH | Float | NULL | Parallel Length |
| COM_SOURCE | Text | NULL | Data Source |
| COM_STANDARD | Json | NULL | Standards Applied |
| COM_DATE | Datetime | CURRENT_TIMESTAMP | Date Added |

### The mechanical properties table records the specimen dimensions, mechanical testing methods, and the force–displacement curves obtained from experiments, along with derived mechanical indicators such as modulus and strength.

## **Table S6. Quantitative summary table for BCC, RPLS, and UML.**

| **Name** | **BCC** | **RPLS** | **UML** |
| --- | --- | --- | --- |
| Load-bearing capacity (kN) | 57.2 | 92.9 | 62.4 |
| Initial yield load (kN) | 38.4 | 73.8 | 39.9 |
| Effective modulus (MPa) | 2347 | 4365 | 4008 |
| Failure mode | Core-centered failure (core region) | Edge-centered failure (away from the core region) | Core-centered failure (core region) |
| Indenter displacement at failure (mm) | 2.81 | 2.36 | 2.34 |

Values are extracted from the force–displacement curves. The failure mode indicates the dominant initiation location observed in the specimen.

# **Supplementary Movies**

## **Movie S1.**

### Stress Distribution during Compression Simulation of a Programmable Component.

## **Movie S2.**

### Compression Simulation of a Uniform BCC Structure.

## **Movie S3.**

### Compression Simulation of a Regionally Programmable RPLS Structure.

## **Movie S4.**

Compression Simulation of a Unifor
